# Evaluation of different genomic regions of rotavirus B and rotavirus C for development of real-time RT‒PCR assays

**DOI:** 10.1186/s12985-024-02369-z

**Published:** 2024-04-24

**Authors:** Madhuri S. Joshi, Manohar S. Shinde, Mallika Lavania

**Affiliations:** Enteric Viruses Group, ICMR- National Institute of Virology, 20-A, Dr. Ambedkar Road. Pune-411 001, Pune, India

**Keywords:** Rotavirus, GBR, GCR, qRT‒PCR, Viral load

## Abstract

**Background:**

The causative agents of diarrhea, rotavirus B (RVB) and rotavirus C (RVC) are common in adults and patients of all age groups, respectively. Due to the Rotavirus A (RVA) vaccination program, a significant decrease in the number of gastroenteritis cases has been observed globally. The replacement of RVA infections with RVB, RVC, or other related serogroups is suspected due to the possibility of reducing natural selective constraints due to RVA infections. The data available on RVB and RVC incidence are scant due to the lack of cheap and rapid commercial diagnostic assays and the focus on RVA infections. The present study aimed to develop real-time RT‒PCR assays using the data from all genomic RNA segments of human RVB and RVC strains available in the Gene Bank.

**Results:**

Among the 11 gene segments, NSP3 and NSP5 of RVB and the VP6 gene of RVC were found to be suitable for real-time RT‒PCR (qRT‒PCR) assays. Fecal specimens collected from diarrheal patients were tested simultaneously for the presence of RVB (*n* = 192) and RVC (*n* = 188) using the respective conventional RT‒PCR and newly developed qRT‒PCR assays. All RVB- and RVC-positive specimens were reactive in their respective qRT‒PCR assays and had Ct values ranging between 23.69 and 41.97 and 11.49 and 36.05, respectively. All known positive and negative specimens for other viral agents were nonreactive, and comparative analysis showed 100% concordance with conventional RT‒PCR assays.

**Conclusions:**

The suitability of the NSP5 gene of RVB and the VP6 gene of RVC was verified via qRT‒PCR assays, which showed 100% sensitivity and specificity. The rapid qRT‒PCR assays developed will be useful diagnostic tools, especially during diarrheal outbreaks for testing non-RVA rotaviral agents and reducing the unnecessary use of antibiotics.

## Introduction

Diarrhea is a global health problem associated with approximately 1.7 to 5 billion cases per year and 19.11% of deaths from diarrhea in 2019 [1–3]. Despite improvements in general sanitation, hygiene, diarrheal treatments, and the use of rotavirus A (RVA) vaccines, diarrhea is a main contributor to childhood mortality, especially in lower- and medium-income countries [4]. A significant decrease in pediatric gastroenteritis cases after RVA vaccination was suspected to reduce natural selective constraints, and replace RVA infections with rotavirus B (RVB), rotavirus C (RVC), or other related serogroups [5]. Recently, dehydration, age, sex-specific vulnerabilities, and causes of diarrhea other than RVA were demonstrated to be important risk factors for childhood diarrheal mortality [4]. Among the non-RVA Rota viral agents, RVB, RVC, and other enteric viruses, namely, noroviruses, adenoviruses, and astroviruses, are the main causative agents.

RVB is known to infect humans and porcine, bovine, murine, and caprine animal species [6–10]. RVB infection with severe watery, cholera-like diarrhea has been documented in large diarrheal epidemics in China, India, Bangladesh, Myanmar, and Nepal [5, 9, 11–15]. The limited studies documented to date have shown the dominance of RVB infections in adults/adolescents, especially in outbreak cases. The occurrence of RVB in diarrheal outbreaks in children [16] and sporadic cases has also been documented [12, 17, 18].

RVC infections are widespread and enzootic in porcine species [19]. The etiological role of human RVC strains has been documented in a small number of sporadic and outbreak cases among all age groups [20–22].

To date, data on diarrhea incidence and etiologies among populations older than five years are scarce [1]. There was a 50% increase in the number of adults older than 70 years (from 1990 to 2016), and nearly three-quarters of diarrhea deaths occurred in individuals older than five years, with a particularly high burden in adults older than 70 years [1]. Identification of diarrhea as a major public health problem among adults/adolescents as well as older individuals highlights the need for studies on diarrheal etiology [1, 23].

On the basis of the scarcity of studies on RVB/RVC, investigations of diarrhea patients in all age groups from different geographical regions are necessary to determine the exact prevalence rate and circulation pattern of these diseases in the human population. To correctly diagnose disease and enable successful prevention and control actions, accurate pathogen identification is essential. Probe-based real-time RT‒PCR (qRT‒PCR) assays, which are known for minimal chances of cross-contamination, are highly sensitive, specific, quick, and useful for quantitative estimation of the viral genome. By adding different fluorophores for each probe, multiplex assays can also be developed for the simultaneous detection of different targets. This study focused on the development of qRT‒PCR assays for the detection of RVB and RVC infections among diarrheal patients.

## Materials and methods

### Designing primers and probes

The suitability of the 11 genes for the detection of RVB and RVC was determined by using nucleotide sequence data retrieved from GenBank (www.ncbi.nlm.nih.gov). Multiple nucleotide sequences obtained were aligned with CLUSTAL W utilizing 24, 26, 23, 44, 32, 63, 23, 92, 23, 32, and 22 sequences (complete or partial) of the VP1, VP2, VP3, VP4, VP6, VP7, NSP1, NSP2, NSP3, NSP4 and NSP5 genes of RVB strains, respectively [24]. The nucleotide sequence data consisted of 119, 60, 84, 37, 38, 37, 39, 40, 39, 56, and 38 strains for the VP7, VP4, VP6, VP1, VP2, VP3, NSP1, NSP2, NSP3, NSP4 and NSP5 genes of human RVC strains, respectively. Primers and probes were designed and finalized using the Primer Quest tool from IDT (https://www.idtdna.com/pages/tools/primerquest) and modified manually. All sets of primers and probes used were examined for self-annealing sites, hairpin loop formation, 3’ complementarities, and melting temperatures (Tm) using the IDT oligonucleotide calculator tool (http://www.idtdna.com/analyzer/Applications/OligoAnalyzer).

### Preparation of the reference RNA standard

A portion of the NSP5 gene (608 bp) of the RVB strain was amplified using the primers RVB_NSP5F (TAA TAC GAC TCA CTA TAG GGA GAG TCAGTAGACGGCTGGAAACGTTG) and RVB_NSP5R (TAA TAC GAC TCA CTA TAG GGA GAG AATATAACTCAAGAGGTTRACCC), with the T7 promoter sequence at the 5’ end of both primers. Similarly, the VP6 gene of RVC (1335 bp) was amplified using the primers RVC_VP6F [TAA TAC GAC TCA CTA TAG GGA GAG CTCATTCACAATGGATGTACTTTTTTC (13–39)] and RVC_VP6R [TAA TAC GAC TCA CTA TAG GGA GAG CATAGTTCACATTTCATCCTTCTGGGGA (1348–1321)]. The amplification of the respective PCR products was confirmed by agarose gel electrophoresis. Amplicons were cleaned by using a Qiagen direct PCR purification kit (Cat No- 28,104 Qiagen, Hilden, Germany). In vitro, transcribed (IVT) RNA was synthesized using a T7 Riboprobe® System (Cat No: P1440; Promega, USA) according to the manufacturer's instructions. The concentration of synthetic fragments of transcribed RNA was measured by fluorometric analysis (Qubit, Thermo Scientific). A standard curve was generated by plotting the log dilution of the RNA transcripts against the Ct value to determine the sensitivity of the assays. The copy number (RNA concentration) was calculated by using the equation Copy number (molecules/µL) = [concentration (ng/µL) × 6.022 × 10^23^ (molecules/mol)]/[length of amplicon X 340 (g/mol) × 10^9^ (ng/g)], as described earlier [25]. To determine the viral loads of RVB and RVC in the fecal specimens, serial tenfold dilutions of RNA transcripts were prepared and used as standards in the assay runs.

### Specimens

Fecal specimens (*n* = 192) collected from Maharashtra, India, were shortlisted for validation of the RVB qRT‒PCR assay. The panel consisted of known positive specimens for RVB (*n* = 31), RVA (*n* = 45), norovirus (*n* = 12), adenovirus (*n* = 15), astrovirus (*n* = 9), and enterovirus (*n* = 5), and negative specimens for all viral agents (*n* = 75) according to methods described previously [15, 26–30]. Similarly, a qRT‒PCR assay for RVC was performed on fecal specimens (*n* = 188) that were positive for RVC (*n* = 8), porcine RVC (*n* = 16), RVA (*n* = 45), RVB (*n* = 5), norovirus (*n* = 12), adenovirus (*n* = 15), astrovirus (*n* = 9), and enterovirus (*n* = 5) and negative for all viral agents (*n* = 73).

### RNA extraction, Conventional RT‒PCR and qRT‒PCR assays

The supernatant of 30% of the fecal suspensions was subjected to RNA extraction using the automated MagMAX Viral/Pathogen Nucleic Acid Isolation Kit (Applied Biosystems, USA) according to the manufacturer’s instructions. Conventional RT‒PCR assays for RVB/RVC detection were used as the gold standard and were carried out as described earlier [15, 22].

The qRT‒PCR assays were carried out using the primers and probes described in Table 1. Briefly, the qRT‒PCR mixture consisted of Invitrogen™ SuperScript™ III Platinum™ One-Step qRT‒PCR master mix (17 µl) (Applied Biosystems, Foster City, CA, USA) containing 0.5 µl of each of the forward and reverse primers as well as the probe (10 µM concentration each) and 8 µl of RNA. The thermal cycling procedure consisted of denaturation for 5 min at 95 °C, cDNA synthesis at 50 °C for 30 min, and RT inactivation at 95 °C for 1 min, followed by 45 cycles of denaturation at 95 °C for 15 s, annealing, and extension at 60 °C for 1 min performed on a CFX96 Real-Time Detection System (Bio-Rad). A sample was considered positive if its sigmoidal amplification curve crossed the threshold line before 40 cycles. A nontemplate control was used in each of the assays. To avoid cross-contamination, RNA was extracted from standard preparations and fecal specimens in separate laboratories.

**Table 1:** Primers and probes designed for RVB and RVC detection

Name	Nucleotide sequence (5’-3’)	Accession number of the referred strain and location of the primer/probe	Amplicon size
**RVB NSP5_16F**	GTAGACGGCTGGAAACGTTG	KU562894.1(16–35)	170 bp
**RVB NSP5_186R**	GAATCAACCAARTCAAYATCAGATT	KU562894.1(186–162)	
**RVB NSP5_60P**	ARTTGAACTCAGACGCTTCTGCCA	KU562894.1(81–60)	
**RVC VP6_322F**	TTGTTAGAGAAGCTTCAAGAAATGG	MN206066.1 (321–345)	146 bp
**RVC VP6_469R**	ACAGGATTCTCACGTCTTGATTG	MN206066.1 (467–446)	
**RVC VP6_348P**	ATGCAACCYCARTCRCCAGCYCTT	MN206066.1 (347–370)	

*F* Forward primer, *R* Reverse primer, *P* Probe, *Y* C/T, *R* A/G

### Statistical analysis

Statistical analysis of the data was performed using One-way ANOVA with Tukey's post hoc test, Student's t test and Pearson's correlation coefficient with the software jamovi v.2.3.26 [31]. Retrieved from https://www.jamovi.org.

## Results

Among the 11 gene segments, conserved nucleotide stretches suitable for designing the primers and probes were observed in NSP3 and NSP5 of RVB and the VP6 gene of RVC. The designed primers and probes were examined for self-annealing sites, hairpin loop formation, 3’ complementarities, and melting temperatures (Tm) using the IDT oligonucleotide calculator tool and found suitable for real-time RT-PCR assay. However, the primers and probes were synthesized for the NSP5 gene of RVB and the VP6 gene of RVC and were labeled with 6-FAM and VIC reporter dyes, respectively, at the 5’ end and evaluated for the detection of RVB and RVC. The GenBank accession numbers of the strains refer to the locations of the primers and probes, and the sizes of the PCR products are described in Table 1.

**Fig. 1:**
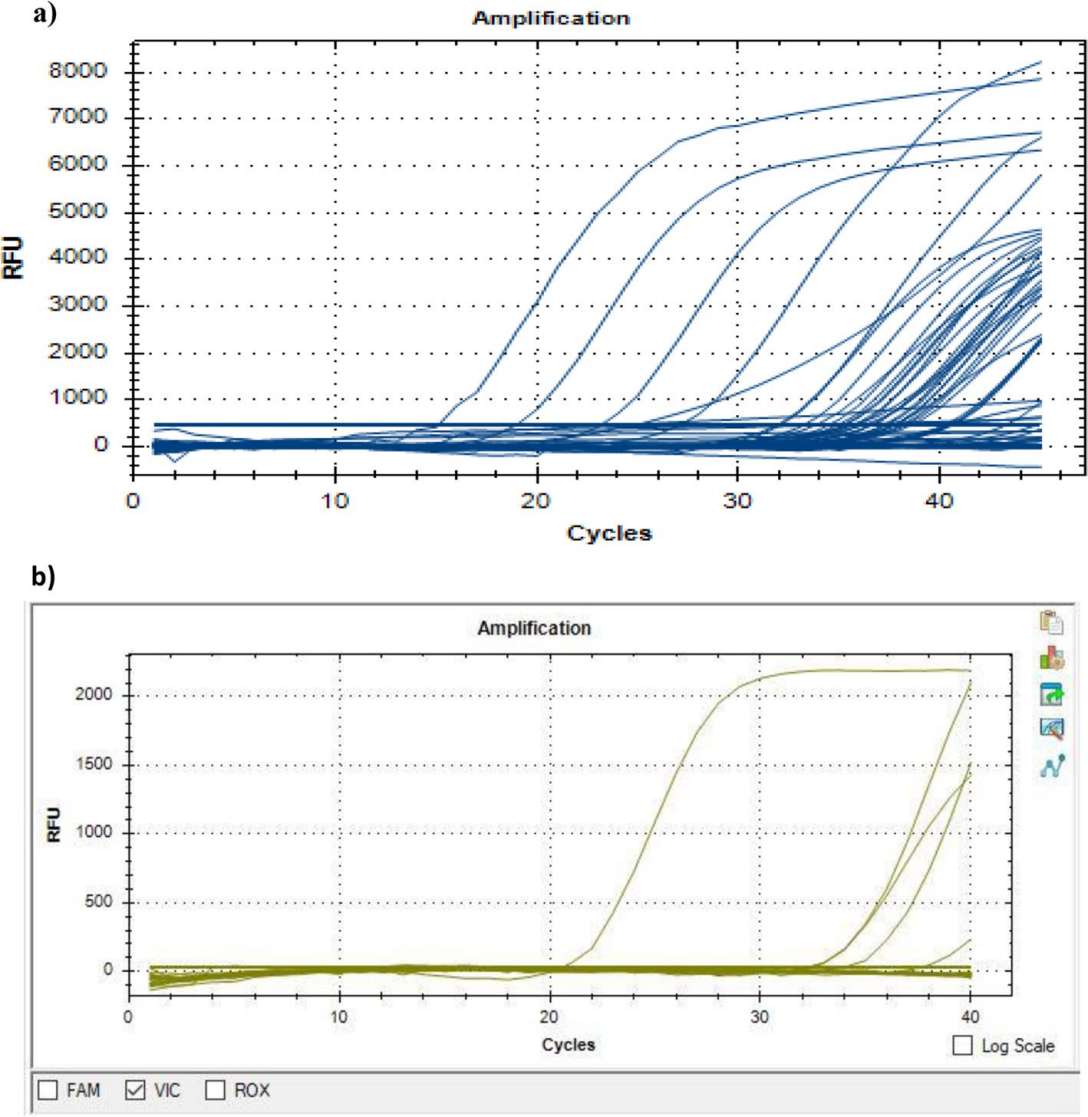
A representative amplification curve of RVB-positive (**a**) and RVC-positive (**b**) specimens determined via qRT‒PCR

Fecal specimens collected from sporadic and outbreak cases of AGE were tested simultaneously for the presence of RVB (*n* = 192) and RVC (*n* = 188) using the respective conventional RT‒PCR and newly developed qRT‒PCR assays. All RVB (*n* = 31) and RVC (*n* = 8) positive specimens were reactive according to the qRT‒PCR assays and presented Ct values ranging between 23.69 and 41.97 and between 11.49 and 36.05, respectively (Fig. 1). All known positive and negative specimens for other viral agents were nonreactive, and comparative analysis showed 100% concordance with conventional RT‒PCR assays.

**Fig. 2:**
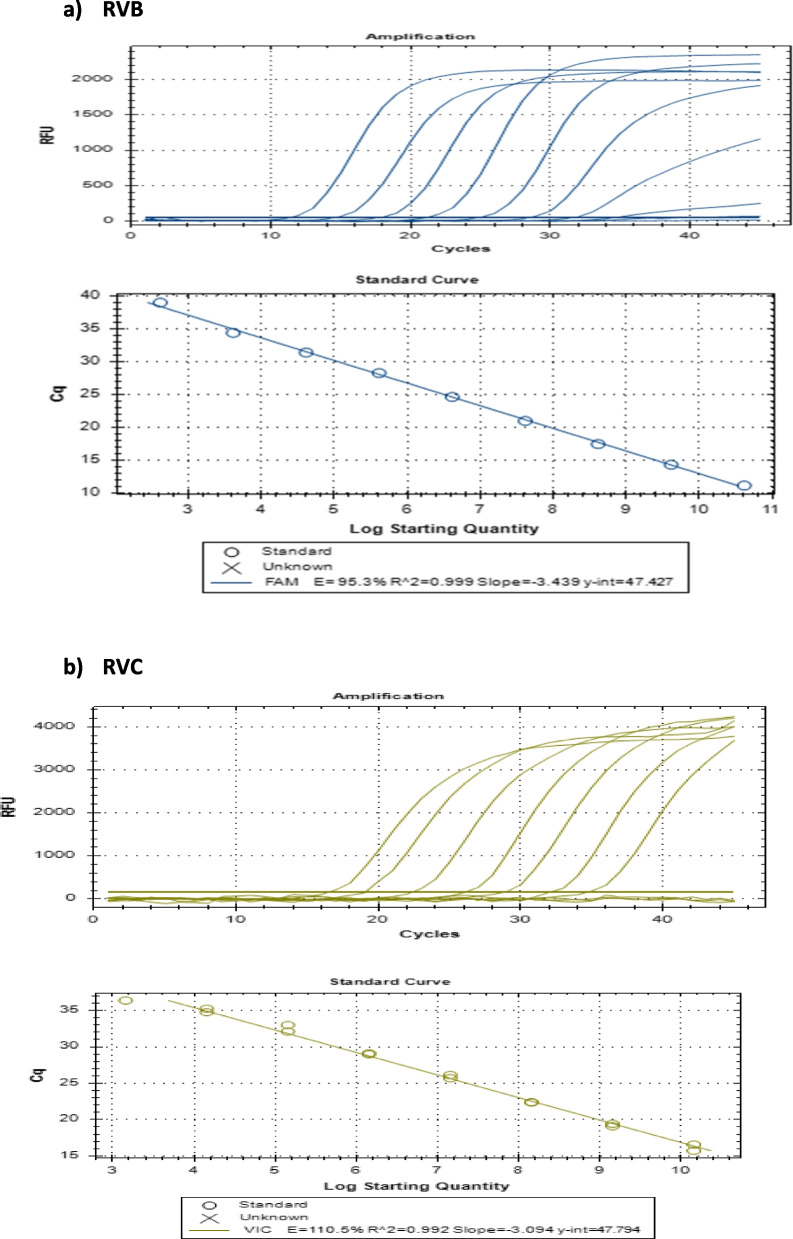
Amplification curves of serial tenfold dilutions of RNA transcripts and the linear relationship between the log transcript copy number and the number of quantitation cycles. The relative fluorescence units on the Y axis refer to the fluorescence emission of a reaction minus the background fluorescence

To determine the efficiency of the qRT‒PCR assays, serial tenfold dilutions (_10_^1^_–10_^9^) of RNA transcripts from RVB/RVC were used as standards in assay runs along with template and nontemplate controls. The plot of Ct values versus log transcript copy number of RVB indicated a linear correlation with an R^2^ value of 0.999, and the efficiency of the assay was calculated to be 95.3% (Fig. 2a). Analysis of the calibration data indicated that the range of the quantitative analysis was from 12.3 × 10^9^ to 12.3 × 10^2^ copies per reaction, corresponding to Ct values of 13.25–37.32. Similarly, after using serial tenfold dilutions (10^1^—10^9^) of the RNA transcripts of RVC, a linear correlation was observed, with R^2^ values of 0.992 and a 110.2% efficiency for the assay (Fig. 2b). Analysis of the calibration data indicated that the range of the quantitative analysis was from 4.3 × 10^9^ to 4.3 × 10^2^ copies per reaction, corresponding to Ct values of 16.65—39.3, respectively.

RVB (*n* = 31) and RVC (*n* = 8) positive specimens presented viral loads between 9.11 × 10^8^ and 3.57 × 10^4^ (Table 2) and between 6.61 × 10^12^ and 2.62 × 10^5^ copies per reaction, respectively. One-way ANOVA with Tukey's post hoc test was performed to compare the severity of the patients with mild (*n* = 3), moderate (*n* = 12), or severe (*n* = 15) disease with the corresponding RVB viral load, and the results were not significantly different. Tukey’s post hoc test showed no significant difference in the mean log viral load of RVB between each pair of severity groups. Student's t test was used to compare the mean log viral load of RVB between females (7.09 ± 1.19) and males (7.13 ± 0.921), and the difference was not significant. Pearson's correlation coefficient (r) between age and the log viral load of RVB was 0.036, which indicated no significant correlation. Due to the small sample size, a similar analysis was not possible for RVC-positive specimens.

**Table 2:** Details of the RVB-positive specimens and virological analysis

SN	Sample IDs	Age (Years)	Gender	Disease Severity	Conventional RT PCR	qRT‒PCR Ct value	Viral Load
1	EVG 001	25	F	Moderate	Pos	36.3	7.87*104
2	EVG 002	23	F	Moderate	Pos	32.3	1.15*106
3	EVG 003	30	M	Moderate	Pos	25.81	2.46*108
4	EVG 004	30	F	Moderate	Pos	31.56	7.01*106
5	EVG 005	32	M	Severe	Pos	26.24	1.58*108
6	EVG 006	32	M	Moderate	Pos	36.12	4.17*105
7	EVG 007	25	M	Severe	Pos	36.12	3.11*105
8	EVG 008	45	M	Severe	Pos	30.51	1.34*107
9	EVG 009	30	F	Severe	Pos	27.24	1.01*108
10	EVG 010	17	F	Severe	Pos	30.35	1.48*107
11	EVG 011	16	F	Severe	Pos	40.09	3.57*104
12	EVG 012	35	F	Severe	Pos	26.23	1.89*108
13	EVG 013	50	F	Severe	Pos	32.02	5.27*106
14	EVG 014	40	F	Severe	Pos	27.65	7.86*107
15	EVG 015	27	F	Severe	Pos	28.63	4.30*107
16	EVG 016	45	F	Severe	Pos	36.16	4.06*105
17	EVG 017	25	F	Severe	Pos	29.74	2.15*107
18	EVG 018	12	F	Severe	Pos	28.79	3.89*107
19	EVG 019	40	F	Severe	Pos	31.77	1.64*106
20	EVG 020	35	F	Severe	Pos	27.26	1.00*108
21	EVG 021	15	M	NA	Pos	31.81	5.99*106
22	EVG 022	32	F	NA	Pos	27.31	9.69*107
23	EVG 023	27	M	NA	Pos	28.76	3.97*107
24	EVG 024	22	M	Mild	Pos	31.76	6.17*106
25	EVG 025	26	F	Moderate	Pos	34.01	1.53*106
26	EVG 026	35	M	Moderate	Pos	28.28	5.34*107
27	EVG 027	35	F	Moderate	Pos	23.69	9.11*108
28	EVG 028	31	F	Moderate	Pos	30.05	1.78*107
29	EVG 029	20	M	Moderate	Pos	30.15	1.68*107
30	EVG 030	14	M	Mild	Pos	30.53	1.32*107
31	EVG 031	13	F	Moderate	Pos	25.17	3.64*108
32	EVG 032	35	M	Moderate	Pos	30.06	1.77*107
33	EVG 033	40	M	Mild	Pos	35.55	5.92*105

*F* Female, *Male* M, *NA* Not available, *Pos* Positive

## Discussion

The evaluation of 11 genomic RNA segments indicated the suitability of the NSP3 and NSP5 genes of the human RVB and the VP6 gene of the human RVC strains for the design of primers and probes. The primers and probes synthesized for the NSP5 (RVB) and VP6 (RVC) genes were validated using conventional RT‒PCR assays and showed 100% concordance. The specificity of the primers and probes was confirmed by testing fecal specimens positive for other enteric viruses as well as specimens negative for all viral agents. The use of qRT‒PCR assays (VP7 gene-based) for the detection of human RVC strains was previously reported [32, 33], but not for RVB strains. Rotaviruses continuously accumulate point mutations and undergo reassortment events [34], therefore, timely evaluation of earlier primers and probes using the strains available thus far is necessary. The sequences of the primers and probes reported earlier were checked using multiple sequence alignment of all the available strains in Genbank (*n* = 23) for the VP7 gene of the RVC. Due to the variable site at the extension site of DNA polymerase or the high number of variable sites or IDT criteria, few of these mutations were found to be unsuitable for qRT‒PCR. The newly developed qRT‒PCR assays displayed 100% sensitivity and specificity when the respective conventional RT‒PCR assays were used as the gold standard.

Analysis of the viral load in the RVB-positive stool specimens revealed no correlation with the severity of the diarrheal disease or age of the patient, and the viral loads were comparable between male and female patients (Table 2). The main limitation of the present study is the small number of RVC-positive specimens available for validation of the assay, and all the RVB-positive specimens included in the study were collected from adult diarrheal patients of two outbreaks reported earlier near Pune city [5, 15]. The absence of RVB in pediatric sporadic cases (hospitalized between 2021 and 2023) observed in the present study is similar to the findings of an earlier study indicating circulation of RVB in the Mumbai and Surat cities of Western India in outbreak cases, with its absence in sporadic cases of gastroenteritis in children [35]. A lower attack rate in children than in adolescents and adults has been documented in multiple outbreaks due to RVB [15, 36–38]. The resistance of the pediatric population to RVB infections was suspected to be due to cross-protection with other serotypes, especially RVA, or may be due to changes in cell receptors and maternal and local immunity [5]. However, the increase in porcine RVC infections in piglets vaccinated with the porcine RVA G5P [8] vaccine documented in Brazil [39] suggests the need for additional studies on non-RVA rotaviral infections.

Sporadic and low rates of infection indicate an evolutionarily stable host‒pathogen relationship, and subtle genetic alterations are suspected to increase pathogenicity, leading to widespread epidemics [40]. The development of a rapid qRT‒PCR assay for the detection of RVB and RVC will be of great help in the future to monitor the role of non-RVA rotaviral agents in sporadic and outbreak cases and, in turn, to reduce the unnecessary use of antibiotics.

## Data Availability

No datasets were generated or analysed during the current study.
